# Continuous exposure to non-lethal doses of sodium iodate induces retinal pigment epithelial cell dysfunction

**DOI:** 10.1038/srep37279

**Published:** 2016-11-16

**Authors:** Xiao-Yu Zhang, Tsz Kin Ng, Mårten Erik Brelén, Di Wu, Jian Xiong Wang, Kwok Ping Chan, Jasmine Sum Yee Yung, Di Cao, Yumeng Wang, Shaodan Zhang, Sun On Chan, Chi Pui Pang

**Affiliations:** 1 Department of Ophthalmology and Visual Sciences, and The Chinese University of Hong Kong, Hong Kong; 2 Department of Ophthalmology, The Fourth People’s Hospital of Shenyang, Shenyang, China; 3 Shenyang Key Laboratory of Ophthalmology, Shenyang, China; 4 School of Biomedical Sciences, The Chinese University of Hong Kong, Hong Kong

## Abstract

Age-related macular degeneration (AMD), characterized by progressive degeneration of retinal pigment epithelium (RPE), is the major cause of irreversible blindness and visual impairment in elderly population. We previously established a RPE degeneration model using an acute high dose sodium iodate to induce oxidative stress. Here we report findings on a prolonged treatment of low doses of sodium iodate on human RPE cells (ARPE-19). RPE cells were treated continuously with low doses (2–10 mM) of sodium iodate for 5 days. Low doses (2–5 mM) of sodium iodate did not reduce RPE cell viability, which is contrasting to cell apoptosis in 10 mM treatment. These low doses are sufficient to retard RPE cell migration and reduced expression of cell junction protein ZO-1. Phagocytotic activity of RPE cells was attenuated by sodium iodate dose-dependently. Sodium iodate also increased expression of FGF-2, but suppressed expression of IL-8, PDGF, TIMP-2 and VEGF. Furthermore, HTRA1 and epithelial-to-mesenchymal transition marker proteins were downregulated, whereas PERK and LC3B-II proteins were upregulated after sodium iodate treatment. These results suggested that prolonged exposure to non-lethal doses of oxidative stress induces RPE cell dysfunctions that resemble conditions in AMD. This model can be used for future drug/treatment investigation on AMD.

Age-related macular degeneration (AMD) is the major cause of irreversible blindness and visual impairment in the elderly population^1^. It is a progressive degenerative disease affecting in particular the macula. AMD can be classified into exudative and non-exudative types, which are characterized by choroidal neovascularization (CNV) and geographic atrophy (GA), respectively^2^. The pathology of GA is characterized by disruption of choriocapillaries and the associated retinal pigment epithelium (RPE) and photoreceptors^3^. RPE under normal conditions plays multiple biological roles that include recycling of bleached visual pigment, maintenance of the inter-photoreceptor matrix and the Bruch membrane, transport of fluids and nutrients between photoreceptors and choriocapillaries and phagocytosis of photoreceptors^4^. During the aging process, RPE cells are reduced, largely by oxidative stress-induced apoptosis^5^. This, together with chronic aberrant inflammation, results in GA.

The etiology of AMD is multi-factorial that includes genetics, inflammation and oxidative stress. We previously identified multiple genetic variants, such as *CFH*, *HTRA1* and *FPR1* genes^6^,^7^,^8^,^9^, associated with AMD, and they could interact additively with oxidative stress-related condition, including cigarette smoking. Moreover, we also identified that HTRA1 expression is related to acute stress^10^, confirming that oxidative stress is an important player in AMD development.

Recently, we have established an animal model of RPE degeneration^11^, in which the RPE and the inner nuclear layer (INL) are damaged selectively by oxidative stress induced by a high dose of sodium iodate^12^. In addition to *in vivo* studies, treatment of human RPE cell line (ARPE-19) with 3000 μg/ml (15.12 mM) sodium iodate for 24 hours can also induce massively cell death, which is not observed in lower doses of sodium iodate (250–1000 μg/ml)^13^. The sodium iodate-induced ARPE-19 cell death *in vitro* has been shown to be associated with increased levels of reactive oxygen species (ROS) and interleukin-8 (IL-8)^14^. Besides, sodium iodate induces necrosis in primary mouse RPE cells with decreased expression of necrostatin-1 (Nec-1)^15^. In addition, acute sodium iodate-induced ARPE-19 cell death is associated with mitochondrial dysfunction and p62 upregulation^16^. While the acute effects of sodium iodate treatment on RPE cells are extensively studied, the effects of a prolonged exposure and the dosage effect of sodium iodate on culture of RPE cells have not been investigated yet.

In AMD pathogenesis, the contribution of oxidative stress is chronic and long lasting, and so results from acute and high dose of oxidative stress might not be relevant to the pathophysiological situation. Other studies have shown that 5 days exposure of 8 mM tert-butylhydroperoxide (TBHP) induces premature senescence in ARPE-19 cells, and rendering the cells become pro-angiogenic^17^. This treatment also upregulates expression of drusen-related molecular chaperones and pro-angiogenic factors^18^. Moreover, exposure of hydrogen peroxide for 1 and 3 days increases the autophagic responses, but decreases in the 14-day treatment^19^. Here we hypothesized that a prolonged exposure of sub-lethal doses of sodium iodate in human RPE cells (ARPE-19), instead of triggering massive cell death as in acute high dose exposure, affects cellular functions in RPE cells that are closely related to pathophysiological conditions of neovascular AMD, which include maintenance of cell integrity, wound healing ability, phagocytotic activity and angiogenic factor expression.

## Results

### Acute and prolonged effects of sodium iodate exposure on RPE cell survival

**Figure 1:**
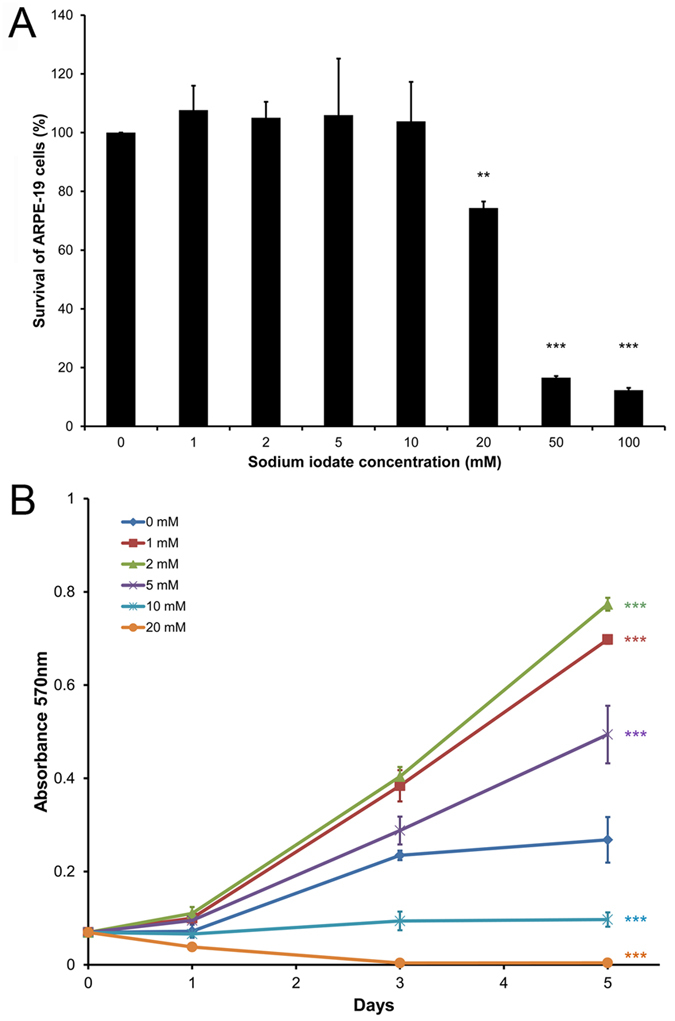
Acute and prolonged effects of sodium iodate exposure on RPE cell survival. Cell viability was assessed by MTT assay. **(A)** ARPE-19 cells were treated with 0–100 mM sodium iodate for 24 hours. Treatment of 20, 50 and 100 mM sodium iodate reduced ARPE-19 cell viability by 25.64%, 83.43% and 87.67%, respectively. Sodium iodate with concentration of 10 mM or below did not significantly cause reduction in RPE cell viability after 24-hour treatment. ‘**’*p* < 0.01; ‘***’*p* < 0.001. **(B) A**RPE-19 cells were treated continuously with 2–10 mM sodium iodate for 5 days. ARPE-19 cell viability was enhanced in 2 and 5 mM treatment groups but reduced in 10 and 20 mM groups compared to the control group (0 mM). ‘***’*p* < 0.001.

Cell viability analyses by MTT (3-(4,5-dimethylthiazol-2-yl)-2,5-diphenyltetrazolium bromide) assay showed that 24-hour treatment of 20, 50 and 100 mM sodium iodate reduced ARPE-19 cell viability by 25.64%, 83.43% and 87.67%, respectively (*p* < 0.01; Fig. 1A). Sodium iodate with concentration of 10 mM or below did not cause significant reduction in RPE cell viability. The results suggested that sodium iodate with concentration of 10 mM or below could be considered as non-lethal doses in acute treatment and would be subjected to further analyses in prolonged treatment conditions.

To determine the prolonged treatment effect, RPE cells were treated daily with 2–10 mM sodium iodate for 5 days, and the effects on cell survival was examined. Sodium iodate at 1, 2 and 5 mM enhanced the viability of ARPE-19 cells by 160.32%, 188.56% and 84.33% at Day 5 when compared to the control, whereas 10 and 20 mM sodium iodate reduced cell viability by 63.81% and 98.51%, respectively (*p* < 0.001; Fig. 1B).

### Dose effect of sodium iodate on RPE cell cycle, cell integrity and mitochondrial oxidative status

**Figure 2:**
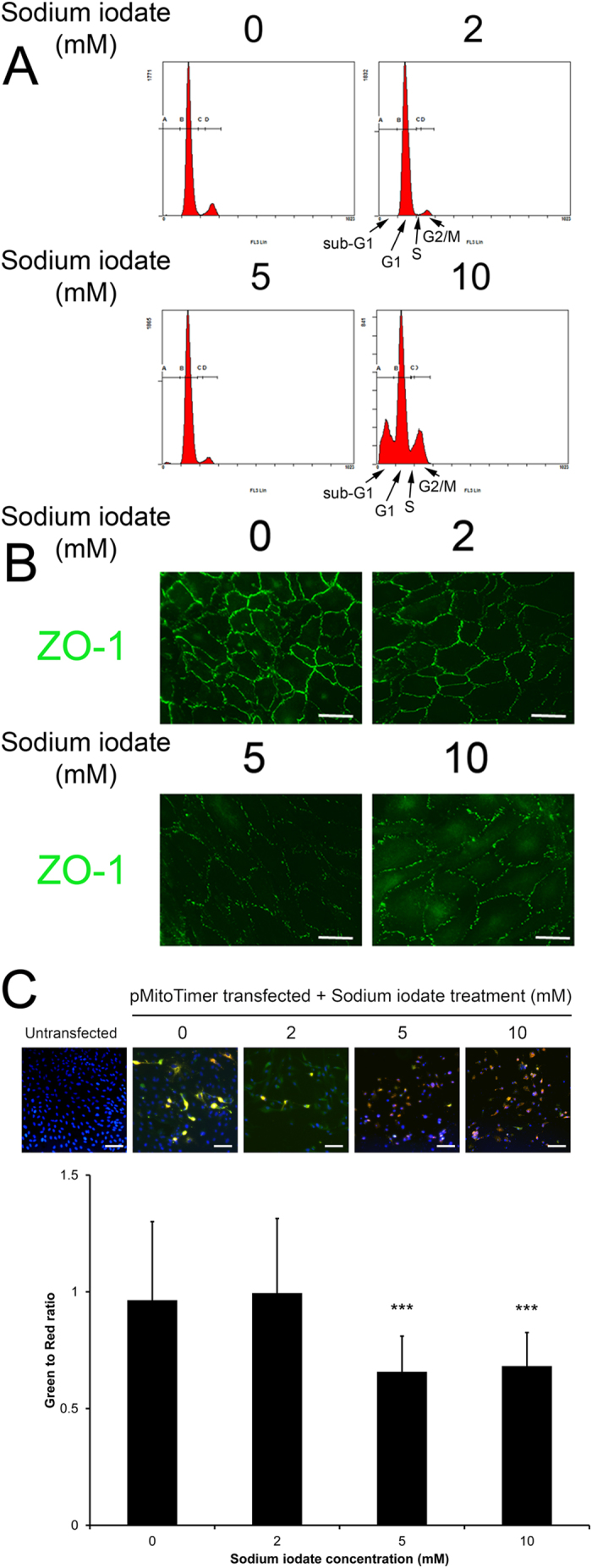
The effect of sodium iodate on RPE cell cycle, cell integrity and mitochondrial oxidative status. (**A**) Cell cycle analysis was evaluated by propidium iodide staining and flow cytometry analysis. There was significant increase in sub-G1 and decrease in G1 populations in the 10 mM treatment group compared to the control group. A: sub-G1 phase; B: G1 phase; C: S phase; D: G2/M phase. **(B)** RPE cell integrity was determined by immunofluorescence analysis of tight junction protein (ZO-1; green). The expression of ZO-1 was dose-dependently inhibited with increasing concentrations of sodium iodate. Scale bars: 50 μm. **(C)** Mitochondrial oxidative stress analysis by MitoTimer reporter in sodium iodate-treated ARPE-19 cells. Fluorescence was shifted from green to red when mitochondria were oxidized. RPE cells with 5 and 10 mM sodium treatments were more oxidized compared to 2 mM and the control group. Blue: DAPI (nuclei). Scale bars: 50 μm. ‘***’*p* < 0.001.

To confirm the results of the cell viability assay, we performed flow cytometry analysis to evaluate apoptosis in APRE-19 cells 5-days after sodium iodate treatment. Similar to the MTT assay, there was a significant increase in apoptotic cells in the sub-G1 population after treatment with 10 mM sodium iodate (26.47 ± 15.52%; *p* < 0.01), as compared to the control (0.99 ± 0.29%; Fig. 2A and Table 1). Treatment with 2 or 5 mM sodium iodate did not show this increase in sub-G1 population (0.58 ± 0.23% and 1.86 ± 1.56%, respectively). These findings indicate that continuous treatment of 10 mM sodium iodate, but not 2 and 5 mM, would cause RPE cell apoptosis. Beside the sub-G1 population, 10 mM treatment group also showed significant difference in G1 population (56.42 ± 6.88%; *p* < 0.01) when compared to the control (82.63 ± 8.34%). Nevertheless, there was no significant difference in S and G2/M populations among different concentrations of sodium iodate when compared to the control group (Table 1).

Besides, we also examined the expression of zonula occludens protein 1 (ZO-1), which is essential to maintain structural integrity of RPE, in ARPE-19 cells after 5-day sodium iodate treatment. Immunofluorescence analysis showed that the cell membrane organization of ZO-1 protein was dose-dependently disrupted from 2–10 mM of sodium iodate treatment (Fig. 2B), suggesting that continuous treatment of sodium iodate would adversely affect cell adhesion property of RPE cells.

Furthermore, we investigated the mitochondrial oxidative status by *MitoTimer* reporter in the RPE cells treated with sodium iodate (Fig. 2C). Fluorescence of the mitochondria-targeted *MitoTimer* reporter protein would be shifted from green to red when oxidized^20^. Our results showed that RPE cells with 5 and 10 mM sodium treatments had lower green-to-red ratio (0.66 ± 0.15 and 0.68 ± 0.14, respectively) than that in the control group and 2 mM treatment group (0.96 ± 0.34 and 0.99 ± 0.32, respectively), indicating that the mitochondria in 5 or 10 mM sodium iodate-treated RPE cells were more oxidized. This also confirmed that sodium iodate induces oxidative stress in RPE cells.

### The effect of sodium iodate on RPE cell migration

**Figure 3:**
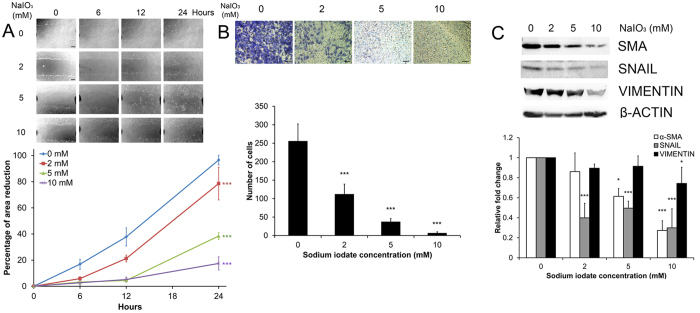
The effect of sodium iodate on RPE cell migration. (**A**) ARPE-19 cell migration after 5-day sodium iodate treatment was evaluated by scratch wound assay. Images were taken at time 0, 6, 12 and 24 hours. The wound area was measured by Image J software. The percentage migration was calculated by the average area reduction at 6, 12 or 24-hour as compared to time 0. The data represented was the mean of triplicated experiments ± standard deviation. Scale bar: 200 μm. ‘***’*p* < 0.001. (**B**) Transwell assay was used to determine the migration of ARPE-19 cells. Bottom membrane of the transwell was stained with 0.2% crystal violet after 5-day treatment of sodium iodate. The numbers of cells migrated to the bottom of the membrane among different treatment groups were counted. Scale bars: 100 μm. ‘***’*p* < 0.001. (**C**) The expression of epithelial-to-mesenchymal transition (EMT) marker protein (α-SMA, SNAIL and VIMENTIN) in ARPE-19 cell after 5-day sodium iodate treatment was evaluated by immunoblotting. Compared to the control group, the expressions of α-SMA, SNAIL and VIMENTIN were significantly reduced in the 5 and 10 mM treatment groups. β-actin was used as housekeeping protein for normalization. ‘*’*p* < 0.05; ‘***’*p* < 0.001. The samples in the gel are run under the same experimental conditions.

RPE cell migration is important in wound healing after injury for recovery and RPE sheet maintenance. Here, the migration of ARPE-19 cells was evaluated by scratch wound assay as well as trans-well assay. The scratch wound was induced after the 5-day sodium iodate treatment. ARPE-19 cell migration was significantly and dose-dependently retarded from 2–10 mM sodium iodate by 21.39%, 61.54% and 82.53%, respectively, at 24 hours, when compared to the control group (*p* < 0.001; Fig. 3A). Similarly, the trans-well assay also demonstrated that a dose dependent reduction in migratory ability of ARPE-19 cells from 2–10 mM of sodium iodate (2 mM: 111.55 ± 27.85 cells/field; 5 mM: 36.75 ± 9.10 cells/field; 10 mM: 6.09 ± 3.92 cells/field), compared to the control group (255.20 ± 47.11 cells/field, *p* < 0.001; Fig. 3B). These analyses indicated that RPE cell migration is inhibited with increasing concentration of sodium iodate.

In order to delineate the mechanism of the retarded cell migration, we determined the protein expression of migration-related epithelial-to-mesenchymal transition (EMT) markers - α-smooth muscle action (α-SMA), SNAIL and VIMENTIN. Immunoblot analyses showed that the expression of α-SMA was significantly reduced in 5 and 10 mM sodium iodate treatment groups by 1.63 and 3.66 folds, respectively (*p* < 0.05), when compared to the control (Fig. 3C). Similarly, the expression of SNAIL was also significantly decreased in all treatment groups by 2.51 (2 mM), 2.02 (5 mM) and 3.34 folds (10 mM; *p* < 0.001). In addition, the expression of VIMENTIN was downregulated only in the 10 mM group by 1.35 folds (*p* < 0.05). The results suggested that the retarded RPE cell migration by sodium iodate was associated with reduced expression of these EMT markers.

### The effect of sodium iodate on angiogenic factor expression in RPE cells

**Figure 4:**
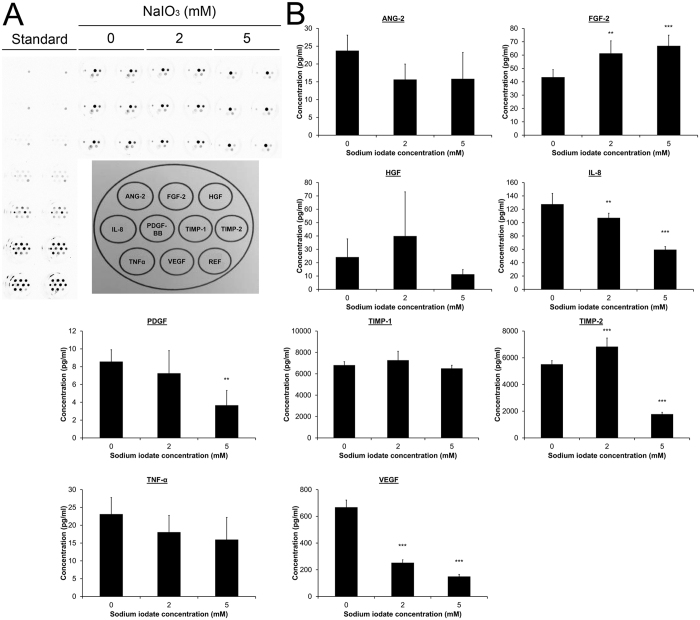
The effect of sodium iodate on angiogenic factor expression in RPE cells. The expression of 9 human angiogenic factors was determined by a multiplex ELISA array. (**A**) The image and topography of the human angiogenic factor multiplex ELISA array. Top (from left to right): ANG-2, FGF-2 and HGF; Middle (from left to right): PDGF, IL-8, TIMP-1 and TIMP-2; Bottom (from left to right): TNFα, VEGF and reference spot. (**B**) Quantification of the expression of the 9 human angiogenic factors according to the standard curves for each factor. The relative expression levels were compared to that of the control group. ‘**’*p* < 0.01; ‘***’*p* < 0.001.

The angiogenic factors secreted by RPE cells, which are critical in the development of CNV, were investigated after treatment with sodium iodate. The supernatant of the cultured ARPE-19 cells 5-day after sodium iodate treatment was collected. 10 mM sodium iodate treatment group was not included since massive cell death affects the number of remaining cells and the secreted proteins in the analysis. The protein expression of 9 angiogenic factors, including angiopoietin-2 (ANG-2), basic fibroblast growth factor (FGF-2), hepatocyte growth factor (HGF), interleukin-8 (IL-8), platelet-derived growth factor (PDGF), TIMP metallopeptidase inhibitor 1 (TIMP-1), TIMP-2, tumor necrosis factor α (TNF-α) and VEGF, were examined by multiplex assays (Fig. 4A). Significant increase in FGF-2 expression was detected in the 2 and 5 mM sodium iodate treatment groups (61.33 ± 9.33 and 66.94 ± 8.00 pg/ml, respectively) compared to the control group (43.50 ± 5.60 pg/ml, *p* < 0.01; Fig. 4B). Moreover, TIMP-2 expression was significantly upregulated in the 2 mM group (6833.68 ± 644.43 pg/ml) but downregulated in the 5 mM group (1781.07 ± 138.10 pg/ml), when compared to the control group (5515.60 ± 264.86 pg/ml; *p* < 0.001). In contrast, IL-8 and VEGF expression was both decreased in 2 and 5 mM groups (IL-8: 107.14 ± 6.89 and 59.56 ± 4.62 pg/ml; VEGF: 252.74 ± 21.75 and 149.82 ± 15.20 pg/ml, respectively), compared to the control (127.64 ± 16.08 and 668.12 ± 53.73 pg/ml; *p* < 0.01). In addition, PDGF expression was also reduced in the 5 mM treatment group (3.67 ± 1.66 pg/ml vs control: 8.58 ± 1.33 pg/ml; *p* < 0.01). These results indicated that oxidative stress-induced RPE cells could promote the proliferation of choroidal endothelial cells through the upregulation of FGF-2^21^.

### The effect of sodium iodate on RPE phagocytotic activity

**Figure 5:**
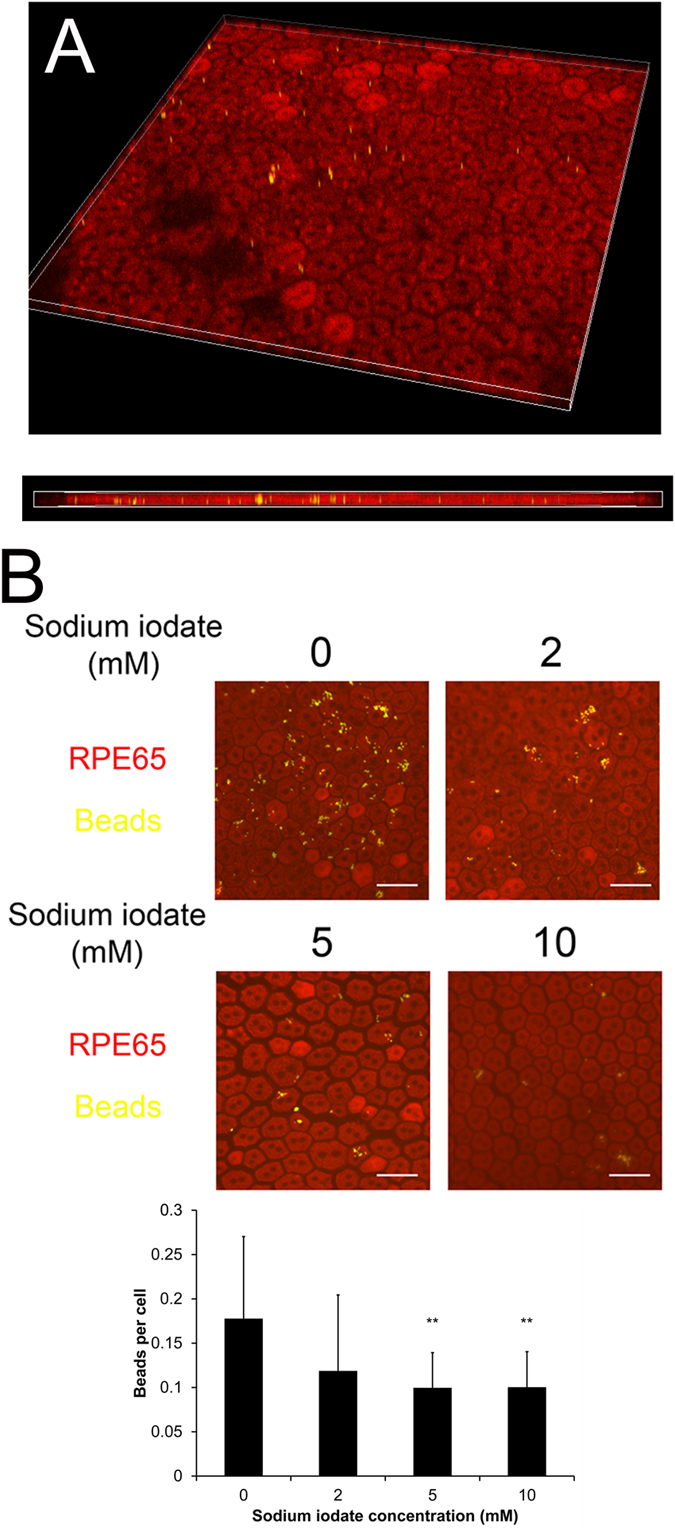
The effect of sodium iodate on RPE phagocytotic activity. Photoreceptor outer segment phagocytosis was performed on the rat RPE explant culture. RPE cells were visualized by the immunofluorescence signal of RPE65 (Red), whereas FITC-labeled latex beads with rat POS (yellow) were opsonized. (**A**) The confocal microscopy image of the phagocytosed, POS opsonized and FITC-labeled latex beads in the RPE65-stained RPE cells. (**B**) After 5-day sodium iodate treatment, the phagocytotic activity of RPE cells was dose-dependently attenuated from 2–10 mM sodium iodate. Scale bars: 50 μm. ‘**’*p* < 0.01

To evaluate the phagocytotic function of RPE cells, we performed the photoreceptor outer segment (POS) phagocytosis analysis on the explant culture of RPE isolated from rats, which preserves the morphology and polarity of native RPE cells. Confocal microscopy imaging confirmed that the FITC-labeled latex beads with POS opsonization were phagocytosed into the RPE65-stained RPE cells (Fig. 5A). Five-days after sodium iodate treatment, the phagocytotic activity of RPE cells on rat POS was dose-dependently attenuated with increasing concentration of sodium iodate (Fig. 5B). The activity was reduced 44.07% with 5 mM sodium iodate treatment (0.099 ± 0.040 beads/cell), whereas 10 mM reduced 43.50% (0.100 ± 0.040 beads/cell) compared to the control (0.177 ± 0.093 beads/cell, *p* < 0.01). Our results demonstrated that a prolonged sodium iodate treatment reduced the phagocytotic activity of RPE cells.

### The effect of sodium iodate on stress-related markers and autophagy related gene expression

**Figure 6:**
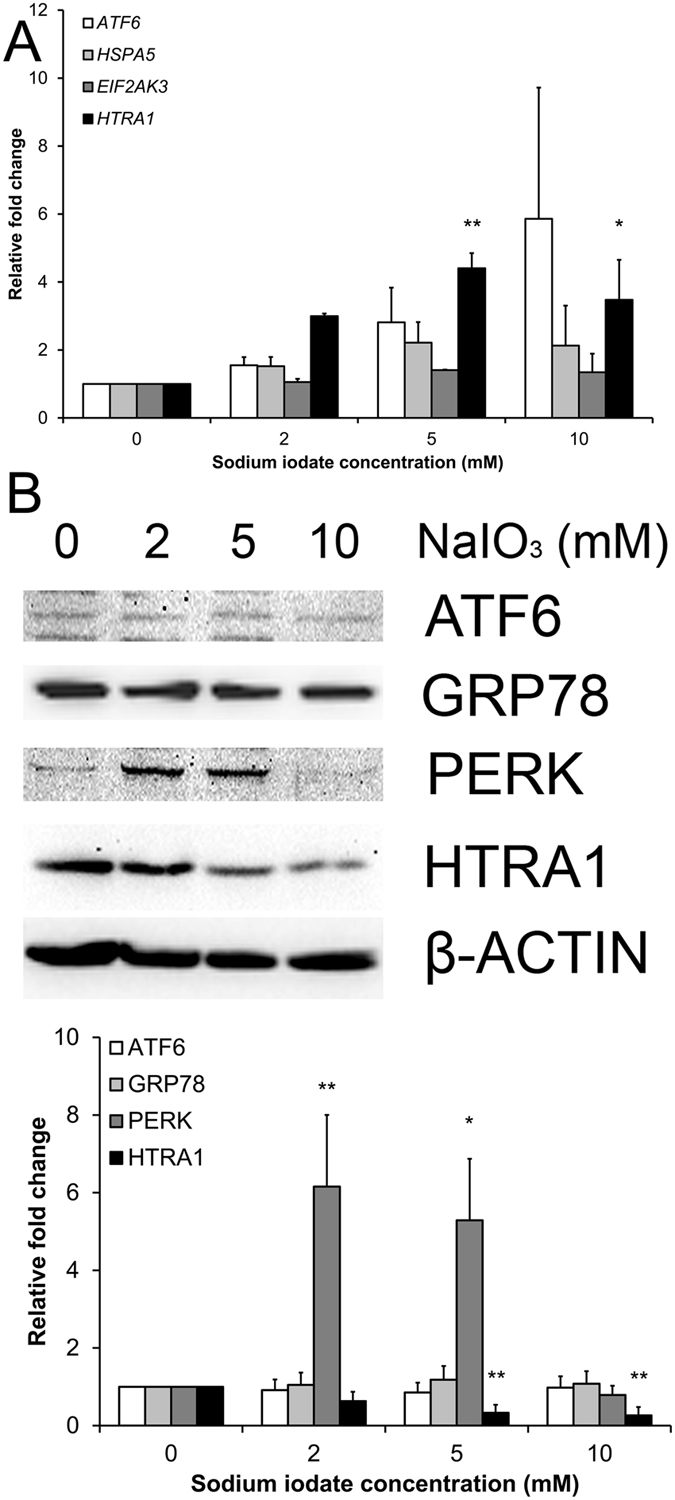
The effect of sodium iodate on ER stress marker expression. The expression of endoplasmic reticulum (ER) stress response gene and protein (GRP78, ATF6, PERK and HTRA1) in ARPE-19 cell after 5-day sodium iodate treatment was evaluated by Sybr green PCR and immunoblotting, respectively. (**A**) Sybr green PCR analysis showed that 5 and 10 mM sodium iodate significantly increased the expression of *HTRA1* gene. *GAPDH* was used as housekeeping gene for normalization. (**B**) Immunoblotting analysis showed that 2 and 5 mM sodium iodate significantly increased the expression of PERK protein, whereas HTRA1 expression was significantly reduced in 5 and 10 mM treatment groups when compared to the control group. β-actin was used as housekeeping protein for normalization. ‘*’*p* < 0.05; ‘**’*p* < 0.01. The samples in the gel are run under the same experimental conditions.

We previously showed that the endoplasmic reticulum (ER) stress is related to RPE cell degeneration^10^. We therefore determined the expression of ER stress markers, such as activating transcription factor 6 (ATF6/*ATF6*), 78 kDa glucose-regulated protein (GRP78/*HSPA5*) and pancreatic ER kinase (PERK/*EIF2AK3*), in ARPE-19 cells after sodium iodate treatment. Sybr green polymerase chain reaction (PCR) analysis demonstrated that *ATF6*, *HSPA5* and *EIF2AK3* did not show statistically significant difference in the sodium iodate treatment groups, compared to the control group (Fig. 6A). Nonetheless, *HTRA1* gene expression was increased in the 5 and 10 mM sodium iodate treatment groups by 4.41 and 3.48 folds respectively, compared to the control group (*p* < 0.05). Immunoblotting analysis showed that 2 and 5 mM sodium iodate significantly increased the expression of PERK by 6.16 and 5.29 folds, respectively (*p* < 0.05; Fig. 6B) when compared to the control group. In contrast, HTRA1 expression was significantly reduced in 5 and 10 mM treatment groups by 3.04 and 3.84 folds, respectively (*p* < 0.01). However, there was no significant difference in ATF6 and GRP78 expression. These findings suggested that sodium iodate induces PERK-driven ER stress response in RPE cells.

**Figure 7:**
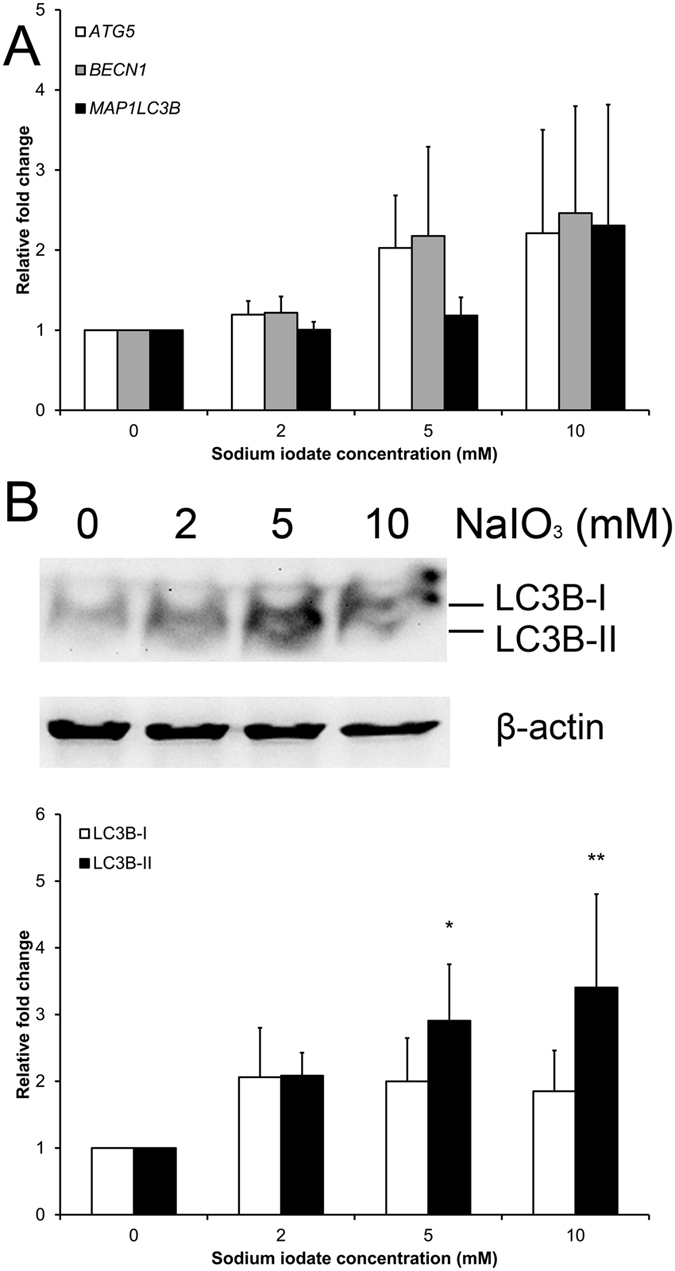
The effect of sodium iodate on autophagy pathway marker expression. The expression of autophagy pathway gene (*ATG5*, *BECN1* and *MAP1LC3B*) as well as LC3B protein in ARPE-19 cells after 5-day sodium iodate treatment was analyzed by Sybr green PCR and immunoblotting, respectively. (**A**) The expressions of *ATG5*, *BECN1* and *MAP1LC3B* genes were not significantly altered among different sodium iodate treatment groups Housekeeping gene (*GAPDH*) was used for normalization. (**B**) The activated form of LC3B protein, LC3B-II, was elevated in 5 and 10 mM of sodium iodate treatment, indicating that sodium iodate activated the autophagy machinery. β-actin was used as housekeeping protein for normalization. The relative expression levels were compared to that of the control group. ‘*’*p* < 0.05; ‘**’*p* < 0.01. The samples in the gel are run under the same experimental conditions.

Apart from the ER stress response, we also examined the gene and protein expression in the autophagy pathway, such as autophagy protein 5 (*ATG5*), Beclin-1 (*BECN1*) and microtubule-associated proteins 1 A/1B light chain 3B (LC3B/*MAP1LC3B*) genes, 5-day after sodium iodate treatment. Sybr green PCR showed that the expressions of *ATG5*, *BECN1* and *MAP1LC3B* genes were not significantly altered in the sodium iodate treatment groups, compared to the control group (Fig. 7A). Nevertheless, the expression of LC3B-II, the activated form of LC3B protein, was dose-dependently elevated with increasing concentration of sodium iodate (5 mM: 2.91 folds; 10 mM: 3.41 folds; *p* < 0.05), compared to the control group (Fig. 7B). The results suggested that autophagy machinery in RPE cells is activated after sodium iodate treatment.

## Discussion

In the current study, our results showed that (1) 5-days prolonged treatment of sodium iodate at 2 and 5 mM do not reduce RPE cell viability, whereas 10 mM treatment reduces RPE viability through apoptosis; (2) prolonged treatment of sodium iodate dose-dependently weakens RPE cell integrity; (3) it also retards RPE cell migration and downregulates EMT marker expression; (4) sodium iodate enhances the expression and secretion of FGF-2 from RPE cells; (5) RPE phagocytotic activity was attenuated dose-dependently by sodium iodate treatment; (6) sodium iodate increases PERK-driven ER stress and autophagy pathway gene expression in RPE cells. Collectively, these data suggest that sodium iodate induces oxidative stress in mitochondria and attenuates the phagocytosis of photoreceptors in RPE cells. Moreover, prolonged oxidative stress also weakens RPE cell-cell adhesion and its wound healing ability for recovery and RPE sheet maintenance so that it could leads to GA as well as proliferation and invasion of choroidal capillaries into the subretinal space^5^, which mimic the pathophysiological conditions in neovascular AMD.

Sodium iodate is a well-known chemical selectively damaging the RPE^11^,^12^. It is a potent oxidant^22^, and could promote the generation of ROS^14^,^23^. Sodium iodate-induced RPE cell death could be related to the upregulation of genes in the cell death, apoptosis and acute response to stress pathways^24^. Moreover, the increased expressions of inflammation-related proteins (complement factor C3 and calcium-independent phospholipase A2)^25^,^26^,^27^, pro-angiogenic factor (IL-8)^14^, chemoattractant (stromal cell-derived factor-1)^25^, oxidative stress-related gene (heme oxygenase)^26^ as well as apoptotic marker (caspase-3)^28^ have been reported. Apart from induction of RPE cell death, sodium iodate would also cause mitochondrial dysfunction and impairment of glycolysis^16^,^29^. In addition, disruption of blood-retina barrier has been indicated in sodium iodate-treated mice^30^,^31^.

In this study, we aimed to characterize the continuous treatment effect of sodium iodate on RPE cells, which is different from previous reports studying the acute effect (24 hours) of sodium iodate on RPE cells^13^,^14^,^15^,^16^.ARPE-19 cell death was observed in 3000 μg/ml (15.12 mM) sodium iodate treatment for 24 hours, but not in lower dose treatment (250–1000 μg/ml; 1.26–5.04 mM)^13^. Coherently, we confirmed that sodium iodate concentration higher than 10 mM would induce significant reduction in RPE cell viability after 24-hour treatment (Fig. 1). Therefore, we chose sodium iodate concentration 10 mM or below to be the acute non-lethal doses for further prolonged treatment analyses.

Similar rationale of prolonged non-lethal oxidative stress has been adopted, in which 24-hour treatment of low doses (200 and 400 μM) of hydrogen peroxide is not sufficient to cause RPE cell death compared to the higher doses (800–1500 μM)^19^. Continuous 14-day treatment of low doses of hydrogen peroxide also does not reduce RPE cell viability (crystal violet assay) but lower the mitochondrial respiration (MTT assay). On the contrary, RPE cells treated with 19.5 mU/ml glucose oxidase (228.6 μM hydrogen peroxide generated) for 2 days reduced MTT signal by 50%^32^. In our current study, continuous 5-day treatment of 2 and 5 mM sodium iodate did not reduce the MTT signal, which was decreased in 10 mM treatment (Fig. 1B). Moreover, 2 and 5 mM sodium iodate did not cause RPE cell apoptosis or morphological change (Fig. 2A and B); therefore, the increased MTT signal could reflect the improved RPE cell viability or mitochondrial activity. The discrepancies among different studies might be due to different effects of different oxidative stress stimuli.

We observed in our study that RPE cell migration was retarded dose-dependently with increasing concentration of sodium iodate (Fig. 3A and B). This is similar to the inhibited migration of endothelial progenitor cells treated with nitroglycerin (vascular oxidative stress inducer)^33^. The retarded migration in RPE cells can be explained by reduced expression of EMT markers, such as α-SMA, SNAIL and VIMENTIN (Fig. 3C). Our observation of reduced EMT marker expression is different from those in cancer cells that chronic oxidative stress would induce malignant transformation as well as increase EMT marker expression^34^. Although we found that the expression of ZO-1 was reduced after sodium iodate treatment (Fig. 2C), this might not be related to increase in EMT in RPE cells. Rather, it would reflect the RPE cell integrity is weakened. Similar ZO-1 loss has been reported in sodium iodate-treated mice^35^. These findings suggest that prolonged oxidative stress weakens RPE cell integrity as well as its ability to repair the wound.

In this study, the expression of PERK in the unfolded protein response pathway was upregulated after the treatment of 2 and 5 mM sodium iodate (Fig. 6). Increased expression of other unfolded protein response pathways, such as CHOP and XBP1, has also been reported in RPE cells with acute sodium iodate treatment^28^. These indicates that sodium iodate would induce ER stress in RPE cells. Apart from ER stress, we observed the upregulation of LC3B-II protein in the autophagy pathway (Fig. 7B). Our results is coherent to the 24-hour, 3.5 mM sodium iodate treatment, which LC3B-II expression is increased in ARPE-19 cells^28^. Similarly, 1 and 3-day hydrogen peroxide treatments also increase the autophagic activity in ARPE-19 cells, but reduces in the 14-day treatment^19^. Increase in autophagic activity has been shown to be associated with the AMD pathology^4^,^36^,^37^. Moreover, the upregulation in autophagy pathway could also be associated with the reduced RPE cell viability (Fig. 1B), which the increase in autophagy can lead to decreased mitochondrial activity by mitophagy in RPE cells^38^, leading to the reduction in the cell viability signal. These indicate that continuous oxidative stress exposure would attenuate RPE cell physiological functions.

Our study showed that continuous treatment of 2 and 5 mM sodium iodate increased FGF-2 expression but decreased IL-8 and VEGF expression (Fig. 4). In contrast, IL-8 expression was found to be increased under 24-hour sodium iodate treatment^14^. Although there is no report on sodium iodate with FGF-2 and VEGF expression, there is a dose dependent increase in FGF-2 expression at non-toxic concentrations of THBP or hydrogen peroxide on ARPE-19 cells^39^, whereas oxidative stress would generally increase the expression of VEGF in RPE cells^40^. Different oxidative stress stimuli and treatment durations might result in different treatment responses.

In summary, it is the first study to characterize the prolonged treatment effect of sodium iodate on RPE cells *in vitro*. Continuous oxidative stress exposure might not instantly affect RPE cell viability or induce cell apoptosis, but it weakens cell integrity, attenuates the ability to repair and reduces phagocytotic activity. Moreover, the expression of EMT markers is decreased, whereas the expressions of FGF-2, PERK and LC3B-II are upregulated. This study revealed the effects and suggested mechanisms of continuous oxidative stress-induced RPE cell dysfunction, which will serve as a platform for further investigation on the drug/treatment effects against AMD.

## Materials and Methods

### Retinal pigment epithelial cell culture

Human RPE cell line (ARPE-19; CRL-2302) from American Type Culture Collection (Manassas, VA) has been established previously^10^,^41^. They were maintained and expanded in Dulbecco’s modified Eagle’s medium and F-12 nutrient mixture (DF-12 culture medium; Gibco BRL, Rockville, MD) supplemented with 10% heat-inactivated fetal bovine serum (Gibco BRL) and 1x penicillin/streptomycin (Gibco BRL) at 37 °C in 5% CO_2_. The cells used for all of the experiments in this study were within 5 passages, and each experiment was repeated at least for 3 times.

### Sodium iodate treatment

Sodium iodate (S4007; Sigma-Aldrich, St. Louis, MO) were dissolved in DF-12 culture medium with the stock concentration of 100 mM. ARPE-19 cells or RPE explants were treated with 1–100 mM of sodium iodate in DF-12 culture medium for 1–5 days. The control group was the cells treated with DF-12 culture medium only. The medium was changed daily in order to maintain a constant level of sodium iodate.

### Cell viability analysis

ARPE-19 cell viability was assessed by MTT assay (Invitrogen, Carlsbad, CA) based on our previously established procedures^42^,^43^. Briefly, 5,000 cells per well were seeded on a 24-well plate (Corning Life Sciences, Lowell, MA) and treated with sodium iodate for 5 days. The analysis (3 wells per sample) was performed on Day 0, 1, 3 and 5. Each sample was incubated with 0.05 mg/ml MTT reagent for 3 hours. After washing out excessive MTT reagent, the purple precipitates were dissolved in isopropanol and transferred to a 96-well plate (Corning Life Sciences) for intensity measurement. The absorbance at wavelength 570 nm with reference 650 nm was measured by a plate reader (Powerwave XS, Bio-Tek Instruments). The percentage of cell viability was determined as OD_570_ sample/OD_570_ control × 100%.

### Cell apoptosis analysis

ARPE-19 cell apoptosis was evaluated by propidium iodide (PI) staining with the flow cytometry analysis. Briefly, 2 × 10^5^ cells were seeded on a 60-mm dish (Corning Life Sciences) and treated with sodium iodate for 5 days. Cells in the supernatant were collected every day when the medium was changed, and they were immediately fixed with 70% ethanol at 4 °C for at least 24 hours. Together with the trypsinized cells at Day 5, the fixed ARPE-19 cells were treated with 50 μl of 100 μg/ml RNase A (Pure link^TM^, Invitrogen) and 950 μl of 50 μg/ml PI (Sigma-Aldrich) in PBS for 20 mins at room temperature in dark. The cells were then passed through a 35 μm cell strainer (Falcon, Corning, NY). 1 × 10^5^ cells for each sample were analyzed by the flow cytometry machine (Cytomics FC500; Beckman Coulter, Indianapolis, IN). Single cells were identified using forward scatter (FS) and side scatter (SS). Cell clumps or doublets were excluded using the pulse area versus the peak height. The distributions of different cell cycle phases were identified using the cell counts (excitation: 488 nm, emission: 620 nm) versus the intensity of PI.

### Cell integrity analysis

ARPE-19 cell integrity was examined by the immunofluorescence analysis of tight junction protein (ZO-1) using our previously established protocol^44^. Briefly, 1 × 10^4^ cells per well were seeded on a glass coverslip in a 12-well plate (Corning Life Sciences). After 5-day sodium iodate treatment, the cells were fixed in 4% paraformaldehyde (Sigma-Aldrich) for 15 min. After permeation and blocking, the treated ARPE-19 cells were labeled with primary mouse monoclonal antibodies against ZO-1 (BD Biosciences, San Jose, CA; Supplementary Table 1) for 18 hours at 4 °C, then with secondary antibody against mouse IgG conjugated with Alexa Fluor^®^488 (Santa Cruz Biotechnology, Dallas, TX) for 1 hour at room temperature. The stained cells were mounted, and the fluorescence signals were visualized under a fluorescence microscope (Eclipse Ni-U; Nikon).

### Mitochondrial oxidative stress analysis

Mitochondrial oxidative status in ARPE-19 cells with sodium iodate treatment was analyzed by the *MitoTimer* reporter assay^20^. Briefly, 1 × 10^5^ cells per dish were seeded on the coverslips in a 24-well plate (Corning Life Sciences) 24 hours before transfection. ARPE-19 cells was transfected with 0.5 μg of *MitoTimer* plasmid DNA, 1 μl P3000 reagent and 1.5 μl Lipofectamine 3000 transfection reagent (Invitrogen) in Opti-MEM medium (Gibco BRL). At 24-hour post-transfection, the culture medium was changed to sodium iodate treatment medium, and the transfected cells were cultured for further 5 days. At the end of the time point, the *MitoTimer*-transfected and sodium iodate-treated RPE cells were fixed with 4% paraformaldehyde (Sigma-Aldrich) at room temperature for 15 mins, and the nuclei were then counter-stained by DAPI. Fluorescence signal of *MitoTimer* was captured and image by a fluorescence microscope (Eclipse Ti, Nikon, Japan). Nine fields were imaged for each coverslip, and 100 cells were counted for each treatment group.

### Cell migration analysis

ARPE-19 cell migration was evaluated by the scratch wound assay. Briefly, 1 × 10^5^ cells per well were seeded on a 12-well plate. After 5-day sodium iodate treatment, scratch wounds were created with 200-μl pipette tips on the pre-seeded confluence cells. The culture was washed after scratch wound induction and replaced by fresh medium. Photomicrographs were taken at time 0 (immediately following the scratch wound), 6, 12 and 24 hours. The wound gaps were measured by ImageJ (version 1.47; NIH, Bethesda, MD). The percentage migration was calculated by the average area reduction at 6, 12 or 24-hour as compared to time 0. Every well have 6 scratch wounds.

Transwell assay was applied to validate the results of the scratch wound assay. Briefly, 200 μl of ARPE-19 cells (1 × 10^5^ cells) was seeded on the upper chamber of a transwell insert and incubated for 10 minutes at 37 °C to allow the cells to settle down. Sodium iodate treatment medium was then added to the lower chamber of a 24-well plate, and incubated for 5 days. Treatment medium in the upper and lower chambers was changed daily. After the 5-day treatment, the medium was replaced by 4% paraformaldehyde for fixation. After 3-time PBS wash, a cotton-tipped applicator was used to carefully remove the PBS and remaining non-migrated cells from the top of the membrane. The transwell membrane was then stained with 0.2% crystal violet at room temperature for 10 min. After PBS washing for three times, the lower chamber was imaged under a phase-contrast microscope. Nine fields were imaged for each well, and each sample was repeated for 3 times.

### Phagocytotic activity analysis

RPE explant culture was used to evaluate the phagocytotic activity of RPE cells *ex vivo*. Briefly, Sprague Dawley (SD) rats, aged 6–8 weeks, were enucleated and dissected. The cornea, iris and lens were first removed. The retina was then separated and collected for the photoreceptor outer segment (POS) processing. The remaining sclera explant, consisting of RPE, Bruch’s membrane, choroid and sclera, was cultured in DF-12 culture medium and treated with sodium iodate for 5 days. All rats were treated according to the guidelines of the ARVO Statement for the Use of Animals in Ophthalmic and Vision Research. The experimental protocol was approved by the Animal Experimentation Ethics Committee of the Chinese University of Hong Kong.

FITC-labeled POS was prepared with the following procedures. Ten retinas from SD rats were dissected and digested in Trypsin-EDTA (Gibco BRL) at 37 °C for 15 min. 1 mg/ml Trypsin inhibitor (Invitrogen) was added and centrifuged for 5 min at 1,500 rpm. The cell pellet was vigorously re-suspended in 5 ml of homogenization buffer (34% sucrose, 65 mM NaCl, 2 mM MgCl_2_) and centrifuged for 4 min at 3,800 rpm. POS was separated from the cell body through vigorous resuspension, and the supernatant was diluted in 10 ml of 10 mM HEPES. The mixture was further centrifuged for 4 min at 3,800 rpm, and the resultant pellet, consisting of the POS fragments, was re-suspended in 1 ml of 10 mM HEPES. The protein concentration was determined by total protein assay (BioRad). 1 mg POS protein was agitated with 25 μl FITC-labelled latex beads (Sigma, 1 μm in diameter) for 1 hour at room temperature. The opsonized beads were washed in 0.9% NaCl twice and re-suspended in 80 μl NaCl.

Phagocytotic activity was assessed after 5-day treatment of sodium iodate. Briefly, the 1 μl of FITC-labeled latex beads with POS opsonization were added to each RPE explant and cultured for 6 hours. The explant was then washed twice with PBS before fixation in 4% paraformaldehyde (Sigma-Aldrich) for overnight at 4 °C. After permeation and blocking, the explant was stained with the primary mouse monoclonal antibody against RPE65 for 18 hours at 4 °C, then with secondary antibody anti-mouse IgG conjugated with Rhodamine RedX (Invitrogen). The stained explant was imaged using a confocal microscope (A1MP, Nikon). Sixteen fields were imaged for each explant, and 200 cells were counted in each field. Phagocytotic activity was quantified by the number of phagocytosed beads per cells.

### Angiogenic factor expression

Angiogenic factor profiling was performed using a multiplex ELISA array (Quansys Biosciences, Logan, UT), which can quantify the expression levels of 9 human angiogenic factors (ANG-2, FGF-2, HGF, PDGF, IL-8, TIMP-1, TIMP-2, TNFα and VEGF). Briefly, 2 × 10^5^ cells were seeded on a 60-mm dish and treated with sodium iodate for 5 days. Supernatant was collected at Day 7, subjected to the multiplex ELISA array, and incubated at room temperature for 1 hour. After washing with the Wash Buffer for 3 times, Detection Mix was added to the plate and incubated at room temperature for 1 hour. After 3-time washing, horse radish peroxidase-conjudated Streptavidin was applied and incubated at room temperature for 15 min. After 6-time washing, the substrate was added, and the plate was imaged immediately using the ChemiDoc^TM^ XRS^+^ system (BioRad, Hercules, CA). The intensities of the spots were quantified and calculated by the Q-View software (Quansys Biosciences) according to the standard curves for each factor. Mean of the 6 repeats for each group was compared for the statistical significance.

### Gene and protein expression analysis

ARPE-19 cells (2 × 10^5^) were seeded on a 60-mm dish and treated with sodium iodate for 5 days. To delineate the mechanism of the sodium iodate effects, the protein expressions of EMT (α-SMA, SNAIL and VIMENTIN), ER stress response (ATF6, GRP78, PERK and HTRA1) and autophagy (LC3B) markers were analyzed by immunoblotting with specific antibodies (Supplementary Table 1), whereas the expressions of ER stress response (*ATF6*, *HSPA5*, *EIF2AK3* and *HTRA1*) and autophagy pathway genes (*ATG5*, *BECN1* and *MAP1LC3B*) were analyzed by Sybr green PCR (Roche) with specific primers (Supplementary Table 2).

For the immunoblotting analysis, the treated cells were lysed by RIPA buffer (Sigma-Aldrich) supplemented with protease and phosphatase inhibitors (Roche). The total protein concentrations of the cell lysates were measured by Protein assay (BioRad). Equal amount of total protein (20 μg) for each denatured samples were resolved on 12.5% SDS-polyacrylamide gel and electro-transferred to nitrocellulose membranes for sequential probing with the primary antibodies and secondary antibodies conjugated with horseradish peroxidase (Santa Cruz Biotechnology). The signals were detected by enhanced chemiluminescence (Amersham Pharmacia, Cleveland, OH) with the ChemiDoc^TM^ XRS^+^ system (BioRad). β-actin was used as housekeeping protein for normalization.

For the gene expression analysis, total RNA was extracted and purified with the TRIzol reagent according to the manufacturer’s protocol (Invitrogen, Carlsbad, CA). 1 μg of total RNA was reverse-transcribed by SuperScript^®^ III reverse transcriptase (Invitrogen). The expression of ER stress response and autophagy pathway genes was evaluated by Sybr green PCR. Housekeeping gene (*GAPDH*) was used for normalization. The relative expression levels were compared to that of the control group.

### Statistical analysis

One-way analysis of variance (ANOVA) with post-hoc Tukey’s test (for multiple testing correction) was used to compare means among different treatment groups. All statistical analyses were performed by commercially available software (IBM SPSS Statistics 22; SPSS Inc., Chicago, IL). Significance was defined as *p* < 0.05.

## Additional Information

**How to cite this article**: Zhang, X.-Y. *et al.* Continuous exposure to non-lethal doses of sodium iodate induces retinal pigment epithelial cell dysfunction. *Sci. Rep.* **6**, 37279; doi: 10.1038/srep37279 (2016).

**Publisher’s note**: Springer Nature remains neutral with regard to jurisdictional claims in published maps and institutional affiliations.

## Supplementary Material

Supplementary Information

## Figures and Tables

**Table 1:** Cell cycle analysis of ARPE-19 cells treated with sodium iodate.

Sodium iodate concentration (mM)	sub-G1 phase	G1 phase	S phase	G2/M phase
0	0.99 ± 0.29%	82.63 ± 8.34%	3.18 ± 1.39%	13.44 ± 7.32%
2	0.58 ± 0.23%	87.96 ± 6.96%	1.51 ± 0.69%	10.00 ± 7.15%
5	1.89 ± 1.56%	85.00 ± 5.88%	2.37 ± 1.89%	10.85 ± 6.80%
10	26.47 ± 15.52%	56.42 ± 6.88%	2.53 ± 0.49%	14.91 ± 8.46%
