# Supplementary material for: Continuous exposure to non-lethal doses of sodium iodate induces retinal pigment epithelial cell dysfunction
Source: Sci Rep. 2016 Nov 16;6:37279. doi: 10.1038/srep37279 (PMC5110957; doi:10.1038/srep37279)
Supplement: Supplementary Information [file srep37279-s1.doc]

**Continuous exposure to non-lethal doses of sodium iodate induces retinal pigment epithelial cell dysfunction**

Xiao-Yu Zhang,^1,2,3,*^ Tsz Kin Ng,^1,*,#^ Mårten Erik Brelén,^1^ Di Wu,^2,3^ Jian Xiong Wang,^1^ Kwok Ping Chan,^1^ Jasmine Sum Yee Yung,^1^ Di Cao,^1^ Yumeng Wang,^1^ Shaodan Zhang,^2,3^ Sun On Chan,^4^ Chi Pui Pang.^1^

^1^ Department of Ophthalmology and Visual Sciences, and ^4^ School of Biomedical Sciences, The Chinese University of Hong Kong, Hong Kong

^2^ Department of Ophthalmology, The Fourth People’s Hospital of Shenyang, and ^3^ Shenyang Key Laboratory of Ophthalmology, Shenyang, China

* Co-first author

^#^**Correspondence:**

Tsz Kin Ng, PhD

Department of Ophthalmology and Visual Sciences, The Chinese University of Hong Kong

4/F, Hong Kong Eye Hospital, 147K Argyle Street, Kowloon, Hong Kong

Phone: +852-39435809; FAX: +852-27159490; E-mail: micntk@hotmail.com

**Supplementary table 1: Antibodies for protein expression analysis.**

| **Protein** | **Company** | **Catalog number** | **Source** | **Dilution factor** |
| --- | --- | --- | --- | --- |
| ZO-1 | BD Biosciences | 610966 | mouse | 1:500 |
| α-SMA | DAKO | M0851 | mouse | 1:500 |
| SNAIL | Abcam | ab180714 | rabbit | 1:500 |
| VIMENTIN | DAKO | M7020 | mouse | 1:1000 |
| GRP78 | BD Biosciences | 610979 | mouse | 1:500 |
| ATF6 | Imgenex | IMG-273 | mouse | 1:500 |
| PERK | Santa Cruz Biotechnology | sc-13073 | rabbit | 1:500 |
| HTRA1 | R&D Biosystems | MAB2916 | mouse | 1:500 |
| RPE65 | Millipore | MAB5428 | mouse | 1:500 |
| LC3B | Cell Signaling Technology | 2775 | rabbit | 1:500 |
| β-ACTIN | Sigma-Aldrich | A3854 | mouse | 1:2000 |

**Supplementary table 2: Primers for gene expression analysis.**

| **Gene** |  | **Primer Sequence (5' > 3')** | **Tm (℃)** | **Accession number** |
| --- | --- | --- | --- | --- |
| *ATG5* | *F:* | GCCATAGCTTGGAGTAGGTTTG | 60 | NM_004849.3 |
|  | *R:* | CGTCCAAACCACACATCTCG |  |  |
| *BECN1* | *F:* | CCGAGGTGAAGAGCATCGG | 60 | NM_003766.4 |
|  | *R:* | TGTGGTAAGTAATGGAGCTGTGA |  |  |
| *MAP1LC3B* | *F:* | TGCGGGCTGAGGAGATACA | 60 | NM_022818.4 |
|  | *R:* | TCTACTCTTTGTTCGAAGGTGCG |  |  |
| *ATF6* | *F:* | TCTCGTCAGCGTTACGGAGTA | 60 | NM_007348.3 |
|  | *R:* | GAGCAGAATCCCAATCTTCATCCA |  |  |
| *HSPA5* | *F:* | CATCAACGAGCCTACGGCA | 60 | NM_005347.4 |
|  | *R:* | TGGCCACAACTTCGAAGACA |  |  |
| *EIF2AK3* | *F:* | TCCGGAACCAGACGATGAGA | 60 | NM_004836.6 |
|  | *R:* | TTCCCAAATACCTCTGGTTTGCTA |  |  |
| *HTRA1* | *F:* | GGAAGCCCGTTTTCCCTTCA | 60 | NM_002775.4 |
|  | *R:* | CAATCACTTCACCGTCCAGGT |  |  |
| *GAPDH* | *F:* | TGTTGCCATCAATGACCCCTT | 60 | NM_002046.3 |
|  | *R:* | CTCCACGACGTACTCAGCG |  |  |
